# Utility of intraoperative pathology consultations of whipple resection specimens and their impact on final margin status

**DOI:** 10.1016/j.heliyon.2023.e20238

**Published:** 2023-09-15

**Authors:** Niloofar Sina, Ekaterina Olkhov-Mitsel, Lina Chen, Paul Karanicolas, Laibao Sun, Preeya Roopchand, Corwyn Rowsell, Tra Truong

**Affiliations:** a Division of Anatomic Pathology, Department of Laboratory Medicine and Molecular Diagnostics, Sunnybrook Health Sciences Centre, Toronto, ON, M4N 3M5, Canada; b Department of Laboratory Medicine & Pathobiology, University of Toronto, Toronto, Ontario, M5S 1A8, Canada; c Department of Surgery, Sunnybrook Health Science Center, Toronto, Ontario, M4N 3M5, Canada; d Department of Laboratory Medicine & Pathobiology, St. Michael's Hospital, Toronto, Ontario, M5B 1W8, Canada

**Keywords:** Intraoperative consults, Frozen section, Pancreatic cancer, Whipple, Pancreaticoduodenectomy, Margins of excision

## Abstract

The resection margin status is a significant surgical prognostic factor for the long-term outcomes of patients undergoing pancreaticoduodenectomy (Whipple procedure). As a result, surgeons frequently rely on intraoperative consults (IOCs) involving frozen sections to evaluate margin clearance during these resections. Nevertheless, the impact of this practice on final margin status and long-term outcomes remains a topic of debate. This study aimed to assess the impact of IOCs on the clearance rate of resection margins following Whipple procedure and distal pancreatectomy. A retrospective database review of all patients who underwent Whipple procedure or distal pancreatectomy at our institution between 2018 and 2020 was performed to evaluate the utility of IOCs by gastrointestinal surgeons and its correlation with final postoperative surgical margin status. A significant variation in the frequency of IOC requests for margins among surgeons was noted. However, the use of frozen section analysis for intraoperative margin assessment was not significantly associated with the clearance rate of final post-operative margins. More frequent use of IOC did not result in higher final margin clearance rate, an important prognostic factor following Whipple procedure.

## 1 Introduction

Pancreatic cancer is the 7th leading cause of cancer-related deaths worldwide, and its prognosis remains poor, with a 5-year survival rate of only 12%, despite aggressive local and systemic treatments [1,2]. The current treatment options for pancreatic cancer include surgical resection in combination with chemotherapy, but relapse rates (both local and distant) are high at 70% [[3], [4], [5]]. The risk of tumour recurrence depends on various variables, including pathological characteristics such as tumour size, stage, grade, lymph node status, and the presence of lymphovascular and perineural invasion, as well as surgical resection margin status [6,7]. Among these prognostic factors, lymph node dissection and resection margin status can be modified surgically. Therefore, intra-operative pathology consultation (IOC) using frozen section for the evaluation of intraoperative margin clearance during pancreaticoduodenectomy (Whipple procedure) and subsequent revision of positive margins is a common surgical practice [8]. The 2021 College of American Pathologists (CAP) Cancer Reporting Protocols state that reported margins for pancreatectomy specimens, including partial, total, and Whipple procedures, should encompass proximal and distal pancreatic parenchymal, pancreatic neck/parenchymal, uncinate (retroperitoneal/superior mesenteric artery), bile duct, proximal (gastric), distal (duodenal/jejunal), among others [9]. Of these, pancreatic and bile duct margins are most frequently requested and most surgically actionable since resection can be extended distally, if frozen section IOC confirms tumour involvement.

Currently, there is a lack of consensus guidelines on the utilization of IOC for Whipple procedures and distal pancreatectomies. Further, several studies have raised questions about the efficacy of IOCs and their potential impact on patient survival [[10], [11], [12], [13], [14]]. Previously published studies have demonstrated that positive pancreatic margins, diagnosed by intra-operative frozen section histology, are associated with positive final post-operative (permanent) resection margins, high risk of recurrence and poor survival [[15], [16], [17]]. Conversely, other studies have suggested that IOC doesn't impact long-term patient survival rates [13,14]. The accuracy of frozen section analysis is subject to variation across studies and relies on the methodology, skill set, and subspecialty of the pathologist [18]. Intraoperative frozen section analysis of tumour margins can present difficulties due to the intricate pathology of pancreatic cancer, inflammation caused by surgical manipulation, and tissue histology artifacts [6]. As a result, there is ongoing debate regarding the utility of IOC for Whipple margins and the re-evaluation of positive findings.

As a part of a quality improvement initiative for IOC practice at our institution, we evaluated the impact of IOC frozen section assessment of Whipple and distal pancreatectomy margins on the final post-operative (permanent) resection margin clearance rate.

## 2 Materials and methods

### 2.1 Study dataset

This quality improvement study was performed at a tertiary-care referral center (Sunnybrook Health Sciences Centre) and was registered as a Quality Improvement project (#229) with the Sunnybrook research ethics office. A retrospective database search of our institutional laboratory information system (Sunquest CoPath) identified patients who underwent pancreatic resection from January 2018 to December 2020. Pancreatic resections included Whipple procedure and distal pancreatectomy (DP). Neoadjuvant therapy was administered to six patients prior to the Whipple procedure. No cases of extended Whipple surgical specimens with vascular resection were included in this cohort. For one case, Whipple procedure was attempted but due to positive pancreatic margin at IOC, resection was extended to total pancreatectomy. Pathology reports were signed out by gastrointestinal (GI) pathologists. Cases that underwent the Whipple procedure or DP and were diagnosed with non-neoplastic disease in their final pathology report were excluded from the study. Duodenal and gastric adenocarcinomas were also excluded.

Specimen grossing was performed according to standardized institutional protocols. Documentation was completed for each case including IOC margin status and final pathology including pathologic stage, tumor location, and margin status for all resection margins. Both the IOC frozen section analysis and final pathological examination, which involved a retrospective review of the same frozen section block and corresponding permanent formalin-fixed, paraffin-embedded block by a second pathologist, were recorded. The status of all other permanent resection margins for each case was also noted. IOC and final margin status were determined based on the final pathology report, with the distance to the closest margin being recorded. The IOC surgical margins were classified as pancreatic margin (PM), bile duct (BD) margins, or other, which included portal vein (PV)/superior mesenteric vein (SMV), superior mesenteric artery (SMA), and gastric margins.

According to the current AJCC (American Joint Committee on Cancer) guidelines, reporting tumor involvement of anterior and non-uncinate posterior surfaces is recommended but not required. Therefore, although pathologists in our institution frequently identify and report tumor involvement of the posterior and/or anterior surface, they were not reported as margins in the final synoptic report.

### 2.2 Categories of surgical resection margin status

Pathology reports were utilized to collect data on frozen section margins. These reports contained information about the intraoperative pathologist consultation diagnosis, as well as the final diagnosis determined by another pathologist after reviewing both the frozen (retrospectively) and permanent (post-operative) sections. Therefore, two pathologists performed the frozen section evaluation of margins. The first IOC pathologist did not participate in the subsequent review of the permanent section. Positive IOC margins were managed according to surgeon preference, after assessing respectability and feasibility. These margins were revised either for intraoperative assessment or assessment on permanent formalin-fixed, paraffin-embedded blocks. The final post-operative (permanent) positive resection margin was defined as invasive cancer < 1 mm from the inked resection margin. False negative IOC margins, which were negative upon frozen section but positive after final pathologic assessment on the same section, were considered to have discordant IOC versus final result.

### 2.3 Statistical analysis

Statistical analysis was performed with SPSS 24.0 (IBM Corp., Armonk, NY, USA). Data correlations were assessed using Chi-Square test and 95% confidence intervals for binary variables. A two-sided p < 0.05 was deemed statistically significant.

## 3 Results

### 3.1 Cohort characteristics

**Fig. 1:**
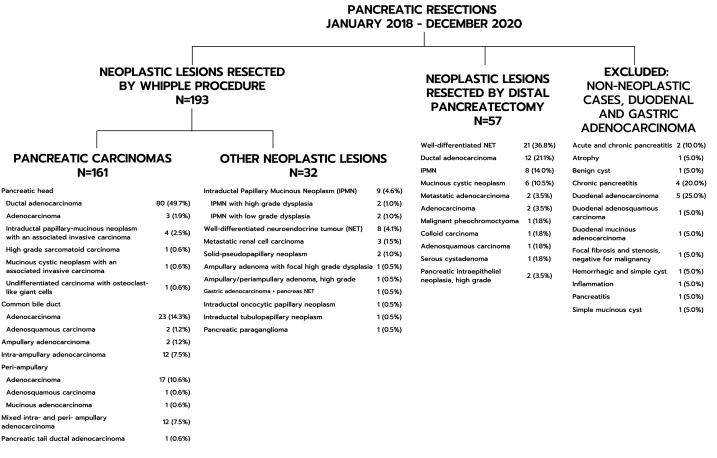
Distribution of histologic types among neoplastic and non-neoplastic lesions that underwent resection via the Whipple procedure and distal pancreatectomy at our institution from January 2018 to December 2020.

During a 3-year period (January 2018-to-December 2020), 193 Whipple and 57 DP procedures were performed at our institution by four pancreatic surgeons for a variety of neoplastic conditions (Fig. 1). In 56.0% (108/193) of Whipple and 14.0% (8/57) of DP specimens, intraoperative frozen section analysis was obtained to assess margins. The pancreatic (n = 95) and bile duct margins (n = 92) were most frequently analyzed, followed by other margins (n = 9). All tumors were resected without leaving macroscopic remnants, 26.4% (51/193) of Whipple and 15.8% (9/57) of DP cases were classified as R1 on the final (permanent) postoperative resection specimen. The final R0-resection rate was 73.6% for Whipple and 84.2% for DP specimens.

**Fig. 2:**
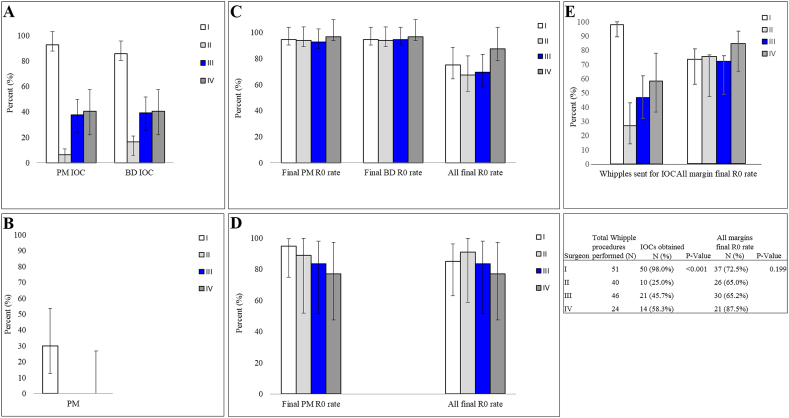
Proportion of IOCs obtained for pancreatic margins (PM) and bile duct margin (BD) assessment during (A) Whipple procedure and (B) distal pancreatectomy for neoplastic lesions by each of four pancreatic surgeons at our institute. Overall final (permanent) margin clearance (R0) rate of (C) Whipple and (D) distal pancreatectomy specimens, including all margin locations. (E) Comparison between the proportion of Whipple cases sent for IOC assessment of margins per surgeons versus their overall clearance rates attained on final pathology reports on the whole cases (all margins) of pancreatic carcinomas. R0, negative margin. *P < 0.05.

Differences in the utilization of IOCs were then investigated among four pancreatic surgeons, revealing a significant disparities in the proportion of IOCs obtained for margin assessment (P < 0.001 for Whipple and P = 0.012 for DP, Fig. 2); surgeon I requested IOCs in 94.6% of their Whipple and 33.3% of their DP cases, while surgeon II requested IOCs in 22.4% and 10.0%, surgeon III in 48.2% and 0%, and surgeon IV in 53.1% and 0% of their Whipple and DP cases, respectively. The overall final margin clearance rate (R0), including all margin sites, for Surgeon I was 75.0% for Whipple and 85.7% for DP, for surgeon II it was 67.3% and 90.0%, for surgeon III it was 69.6% and 84.6%, and for surgeon IV it was 87.5% and 76.9% for Whipple and DP, respectively. There was no significant difference in overall final R0 clearance rate among the surgeons (P = 0.197 for Whipple and P = 0.847 for DP, Fig. 2C and D), despite significant variation in IOC utilization (Fig. 2A and B).

### 3.2 Intraoperative consultation during whipple procedure of malignant resection specimens

Of the 193 Whipple cases in this study, 161 were of malignant lesions which were selected for further assessment. All tumors were documented using the CAP synoptic report template for resection of pancreatic carcinomas. The identified types of malignancies comprised ductal adenocarcinoma (n = 81, 42.0%), adenocarcinoma NOS (n = 50, 25.9%), biliary type and intestinal type adenocarcinomas (n = 39, 20.2%), adenosquamous carcinoma (n = 3, 1.6%), undifferentiated carcinoma (n = 1, 0.5%), mucinous adenocarcinoma (n = 1, 0.5%), mucinous cystic neoplasm with an associated invasive carcinoma (n = 1, 0.5%) intraductal papillary mucinous neoplasm (IPMN) associated with invasive carcinoma (n = 4, 2.1%) and high-grade sarcomatoid carcinoma (n = 1, 0.5%) (Fig. 1). For 95 of these cases (59.0%) IOC on one or more margins was obtained (Group 1), while 66 cases (41.0%) did not have IOC performed (Group 2). Tumors located in the head of the pancreas (57/95, 60.0%) or common bile ducts (19/95, 20.0%) were more frequently subjected to IOC for margin assessment than those in other regions (Table 1). For cases in Group 1, IOC was performed on 78 PM, 84 BD, and 7 other margins.

**Table 1:** Characteristics of 161 pancreatic carcinomas resected by Whipple procedure with IOC (Group 1) or without IOC (Group 2) stratified by sites, pathologic tumor stage (pT) and final status of all margins per case.

	Total	Group 1	Group 2	Chi-square P-Value
	N = 161	N = 95	N = 66	
	N (%)	N (%)	N (%)	
**Tumor type**				
**Pancreatic head**	90 (55.9%)	57 (60.0%)	33 (50.0%)	
Ductal adenocarcinoma	80 (88.9)	50 (87.7)	30 (90.9)	
Adenocarcinoma	3 (3.3)	2 (3.5)	1 (3.0)	
IPMN with an associated invasive carcinoma	4 (4.4)	2 (3.5)	2 (6.1)	
High grade sarcomatoid carcinoma	1 (1.1)	1 (1.8)	0	
MCN with an associated invasive carcinoma	1 (1.1)	1 (1.8)	0	
Undifferentiated carcinoma with osteoclast-like giant cells	1 (1.1)	1 (1.8)	0	
**Common bile duct**	25 (15.5%)	19 (20.0%)	6 (9.1%)	
Adenocarcinoma	23 (92.0)	18 (94.7)	5 (83.3)	
Adenosquamous carcinoma	2 (8.0)	1 (5.3)	1 (16.7)	
Ampullary adenocarcinoma	2 (1.2%)	2 (2.1%)	0	
Intra-ampullary adenocarcinoma	12 (7.5%)	5 (5.3%)	7 (10.6%)	
**Peri-ampullary**	19 (11.8%)	6 (6.3%)	13 (19.7%)	
Adenocarcinoma	17 (89.5)	6 (100)	11 (84.6)	
Adenosquamous carcinoma	1 (5.3)	0	1 (7.7)	
Mucinous adenocarcinoma	1 (5.3)	0	1 (7.7)	
**Mixed intra- and peri- ampullary adenocarcinoma**	12 (7.5%)	6 (6.3%)	6 (9.1%)	
**Pancreatic tail ductal adenocarcinoma**	1 (0.6%)	0	1 (1.5%)	
**pathologic stage (pT)**				0.103
pT1	18 (11.2%)	7 (7.4%)	11 (16.7%)	
pT2	73 (45.3%)	48 (50.5%)	25 (37.9%)	
pT3	69 (42.9%)	39 (41.1%)	30 (45.5%)	
N/A	1 (0.6%)	1 (1.1%)	0	
**IOC margin status**				
Negative		79 (83.2%)		
Positive		16 (16.5%)		
**Final (permanent) margin status across all margin locations**				0.733
Negative	114 (70. 8%)	68 (71. 6%)	46 (69.7%)	
Positive	47 (29.2%)	27 (28.4%)	20 (30.3%)	

*IPMN, Intraductal papillary-mucinous neoplasm; MCN, Mucinous cystic neoplasm.

Pathologic staging (pT) for the study cohort is listed in Table 1; 73/161 (45.3%) of cases were pT2, 69/161 (42.9%) pT3, and 18 (11.2%) cases were pT1. For one case pT could not be determined, thus, it was excluded from analysis. Within Group 1 the pT distribution was pT1 (n = 7, 7.4%), pT2 (n = 48, 50.5%) and pT3 (n = 39, 41.1%). In Group 2 pT1 (n = 11, 16.7%), pT2 (n = 25, 37.9%) and pT3 (n = 30, 45.5%). There were no statistically significant differences in pT distribution between the cases that were sent for IOC (Group 1) and the ones that were not (P = 0.103).

Comparing IOC rates between surgeons, there was a statistically significant difference in the proportion of cases sent for IOC (p < 0.001, Fig. 2E): Surgeon I sent 98.0% (50/51) of their cases for IOC including 49 IOC requests on PM, 45 IOC requests on BD and 7 IOC requests on other margins. Surgeon II sent 25.0% (10/40) of their cases for IOC including 3 PM and 7 BD. Surgeon III sent 45.7% (21/46) of their cases for IOC assessment, including 16 PM and 19 BD. Lastly, surgeon IV sent 58.3% (14/24) of their cases for IOC, including 10 on PM and 13 on BD margins.

Final (permanent) R1 margins (including all margin sites) were determined in 47 (29.2%) of 161 carcinoma cases surgically treated by Whipple procedure. The locations of the positive margins are indicated in Fig. 2E. Of the four pancreatic surgeons, Surgeon I final R0 resection clearance rate was 72.5%, Surgeon II was 65.0%, Surgeon III was 65.2%, Surgeon IV was 87.5%. There was no significant difference in the proportion of overall final (permanent) R0 resection clearance among surgeons (p = 0.199, Fig. 2E), regardless of margin location. This is despite the significant difference in the proportion of cases sent for IOC among the surgeons noted above.

Among the six patients who received neoadjuvant therapy prior to the Whipple procedure, IOC was performed for four individuals, including the assessment of PM and BD margins, all of which were negative for carcinoma. However, upon examination of the permanent (final) sections, one of these cases had a positive SMV margin, while another case had a positive uncinate margin, which were not analyzed on frozen section.

### 3.3 Correlation of matched frozen and permanent section evaluation of margin status

A total of 95 individual, matched pairs of frozen and permanent (final) section margins were analyzed. Comparison of the two diagnostic modalities found discordances in 3 (3.2%) pairs. In 2 pairs, frozen section was reported as benign, but evaluation of the matched permanent section of the same margin block disclosed a carcinoma. In the remaining pair, frozen section was reported as negative for adenocarcinoma but atypical infiltrating epithelioid cells, favoring neuroendocrine neoplasia (NET) were noted. Retrospective re-review of the frozen section by another pathologist as part of final pathologic diagnosis found no evidence of NET or adenocarcinoma at margin. This finding was in agreement with the original IOC review indicating that the frozen section was negative for adenocarcinoma. Therefore, the concordance rate between matched frozen sections and permanent (final) section specimen for the same block was 97.9%.

Frozen section margins were positive in 16 (16.8%) of 95 IOC frozen sections. Surgical revision of margins was attempted in 9 of these cases; tumor persistence was present in 4 cases (2 PM and 2 BD margins) while 5 cases were converted to negative margins post-revision. However, one of these cases had a positive SMV margin on permanent (final) section, which was not analyzed on frozen section (Table 2).

**Table 2:** Characteristics of 16 Cases with positive intraoperative frozen section margins, including information on attempted surgical revision, final permanent margin status, and status of other margins.

Case	Intra-Operative Frozen Section			Surgical revision attempted^a^	Final Permanent Section					
	Margins evaluated				Margins evaluated					
	PM	BD	Other		PM	BD	Uncinate	Proximal	Distal	Other
1	+	–		yes	–	–	–	–	–	
2	+	–		yes	–	–	–	–	–	
3	+			yes	–	–	–	–	–	
4	+	+		yes	–	–	–	–	–	
5		+		yes	–	+	–	–	–	Circumferential margin of CBD (+)
6	–	+	“LBD” (unspecified) (+)	No	–	+	–	–	–	
7	–	–	PV (+), hepatic duct margin (+)	No	–	–	–	–	–	PV (+)
8	–	–	SMA (+)	No	–	–	+	–	–	SMA (+)
9	**+**			No	+	–	–	–	–	SMV (+)
10	+	–		yes	–	–	–	–	–	SMV (+)
11	+	–		yes	+	–	–	–	–	
12	+	–		yes	+	–	–	–	–	
13	–	+		yes	–	+	–	–	–	
14		+		No	–	+	–	–	–	
15		+		No	–	+	–	–	–	
16	–	+		No	–	+	–	–	–	

PM, pancreatic margin; BD, bile duct margin; CBD, common bile duct margin; PV, portal vein margin; SMA, superior mesenteric artery; SMV, superior mesenteric vein margin.

a Including margins that were surgically revised either for further intraoperative assessment or assessment on permanent formalin-fixed, paraffin-embedded blocks.

### 3.4 Comparison of intraoperative consultation versus final permanent pathology results for resection specimens

**Fig. 3:**
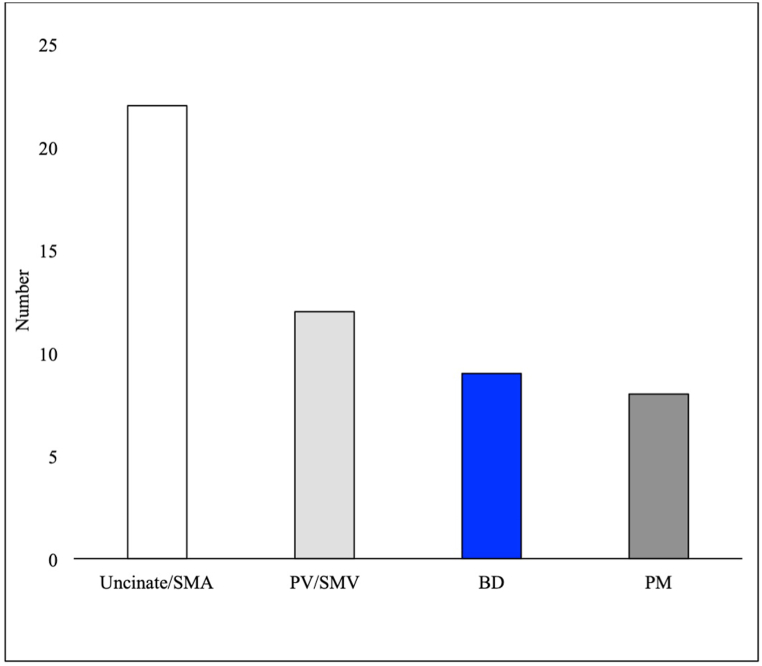
Number of different final (permanent) positive margins reported on Whipple specimens for pancreatic carcinomas. SMV, superior mesenteric vein; PV, portal vein; SMA, superior mesenteric artery; BD, bile duct margin; PM, pancreatic margin.

Of 161 Whipple resection specimens of malignant lesions, 114 (70.8%) were R0 on permanent final section. There was no significant difference in overall final clearance rate (R0) for the entire specimen between non-matched cases that had margins intraoperatively evaluated (Group 1, 68/95, 71.6%) versus those that did not (Group 2, 46/66, 69.7%, p = 0.796), regardless of margin location (Table 1). The most common final (permanent) positive margins reported on all cases were uncinate process/SMA (n = 22), PV/SMV (n = 12), BD (n = 9) and PM (n = 8), as demonstrated in Fig. 3.

## 4 Discussion

The present study aimed to expand on existing literature concerning the impact of IOCs during pancreatic surgery on final post-operative margin status, an important prognostic factor following Whipple procedure. To our knowledge, it is the first study to investigate IOC frozen section utilization during pancreatic surgeries among surgeons within the same institution. The most commonly obtained IOCs during the Whipple procedure for malignant lesions at our institution were PM and BD margins, which is in agreement with the common practice reported in other institutions [19]. Although surgical revision of a positive IOC PM and/or BD can be achieved, other margins may still be involved with the tumor, such as SMV or SMA, which are difficult to revise without exposing patients to additional risks. In fact, the most common final positive margins in our cohort were PV/SMV and uncinate/SMA.

According to our results, a considerable portion of cases (6/7, 85.7%) with positive BD margins on IOC were not revised or remained positive post-revision on final assessment. Thus, our findings suggest that requesting BD margins for IOC assessment may not have significant clinical significance. Most cases with positive PM margins (5/8, 62.5%) achieved negative final margins after revision. Nonetheless, seven of these eight revised cases are ductal adenocarcinomas and previous publications have demonstrated that such revision does not enhance the overall survival of patients [20].

There was no significant difference in pT distribution between the cases that were sent for IOC (Group 1) and the ones that were not (Group 2) in our study. Thus, the surgeon's decision to obtain IOC was not based on preoperative radiologic assessment of tumour size (2.0 cm to >4.0 cm). Further, given the high (97.9%) concordance rate between matched frozen sections and permanent (final) section margin status in our cohort, it was also not likely a factor in the surgeon's decision to negate against requesting margin IOCs. Notably, previous reports indicate that GI pathologists have higher accuracy rates for intraoperative assessment of Whipple margins for pancreatic adenocarcinomas compared to general pathologists (93.5% vs 85.1%) [18]. The concordance rate for IOC and final margins in our study is above this reported average and is probably related to the fact that most IOCs for gastrointestinal cases at our institution are prospectively reviewed by GI pathologists.

In our study, there was a substantial variability observed among surgeons regarding the request for IOC to assess margins during pancreatic surgeries. This variability could potentially stem from the differences in personal experience, training background, preferences, and resources available at the institution of practice. However, we noted that despite this variation, there was no significant difference observed in the overall postoperative final R0 rate (which included all margins) for all neoplastic lesions examined, and for pancreatic carcinomas resected with the Whipple procedure, in particular.

In our cohort, the rate of final (permanent) R1 margin involvement following Whipple procedure for carcinoma cases was 28.8% and 17.2% following DP. Previously reported R1 rates for Whipple specimens vary markedly in the literature from 16% to >75% [19]. Similarly, the reported R1 rates for DP range widely, from <10 to >80% [20]. The R1 rates in our study are consistent with recently published findings by Li B et al. [21] that reported an R1 rate of 31.8% for Whipple procedure in their cohort of 192 patients and a study by Paye F et al. [22] that reported a R1 resection rate of 25.2% for DP of pancreatic adenocarcinomas. This variability can be attributed to heterogeneity in study cohorts as well as the marked divergence in pathology examination practices among different institutions.

Our analysis revealed that there was no significant difference in the overall clearance rate for all margins between cases that underwent IOC on PM and/or BD margins versus cases that did not undergo frozen section assessment of margins. This intriguing finding may be attributable to surgeons' attempts to achieve negative margins in their initial surgical intervention. Additionally, for advanced cases, achieving negative margins, either at first attempt or revision following IOC, may not be feasible [21].

Inconsistency in definitions, nomenclature and grossing techniques of Whipple specimens have been frequently reported and have previously caused confusion in the correlation between clinical and pathologic data [6,10,18,23,24]. Our data was gathered from one academic institute following the same guidelines, which helped to ensure more consistent findings. However, we still encountered some discrepancies in the nomenclature used in reports, especially for localizing the site of tumor origin. The complex nature of Whipple specimens and the three-year time span of reports were two factors that may have contributed to this.

The limitations of our study include a limited number of cases from a single institution, the retrospective nature of the study, lack of clinical and radiologic history and follow up data. Pre-operative information about tumour and lymph node status of each case were not included. The individual experience level of the surgeons involved was not available. Furthermore, there was variability in the definition of R1. Many studies have reported such inconsistencies in reporting positive margins, and many have shown that more comprehensive sectioning of Whipple specimens will raise the rate of R1, especially in retroperitoneal margins such as SMA margin [1,25,26]. Inconsistencies have also been reported on evaluation of R status of pancreatic cancer following neoadjuvant therapy, which may have impacted R1 rate in this study cohort as it included a small number of neoadjuvantly treated patients. Our study focused on the impact of IOCs for Whipple margins on final clearance of the whole specimens. A larger multi-institutional study with higher number of cases including clinical, radiological correlation, and survival analysis is necessary for further investigation of this important aspect of the management of patients with pancreatic adenocarcinoma.

## 5 Conclusion

Our retrospective analysis of 161 cases of Whipple surgery for malignant tumors performed at our institution between January 2018 and December 2020 revealed that, from a surgical pathology standpoint, IOC margin assessment did not result in a higher final (permanent) postoperative R0 rate for resections. These findings support an increasing body of evidence questioning whether 10.13039/100008914IOC during Whipple surgeries performed for adenocarcinomas improves long-term patient outcomes or reduces the risk of R1. The notable variation in the frequency of IOC requests for margins among surgeons suggests the need for development or redefining of guidelines for IOCs during pancreatic surgeries.

## Funding/support

This research did not receive any specific grant from funding agencies in the public, commercial, or not-for-profit sectors.

* The currently submitted manuscript represents original research that was presented, in part, at the 34th European Congress of Pathology held September 3–7,2022 in Basel, Switzerland and the United states and Canadian Academy of Pathology Annual Meeting held March 11–16, 2023 in New Orleans, LA.

## Ethics statement

This study was registered as a Quality Improvement project (#229) with the Sunnybrook research ethics office.

## Funding

This research did not receive any specific grant from funding agencies in the public, commercial, or not-for-profit sectors.

## Author contribution statement

Niloofar Sina: Performed the experiments; Analyzed and interpreted the data; Wrote the paper. Ekaterina Olkhov-Mitsel: Analyzed and interpreted the data; Contributed reagents, materials, analysis tools or data; Wrote the paper. Lina Chen: Analyzed and interpreted the data. Paul Karanicolas: Conceived and designed the experiments; Performed the experiments; Analyzed and interpreted the data. Laibao Sun and Preeya Roopchand: Contributed reagents, materials, analysis tools or data. Corwyn Rowsell: Conceived and designed the experiments. Tra Truong: Conceived and designed the experiments; Performed the experiments; Analyzed and interpreted the data; Wrote the paper.

## Data availability statement

Data will be made available on request.

## Declaration of competing interest

The authors declare that they have no known competing financial interests or personal relationships that could have appeared to influence the work reported in this paper.
